# Supplementary material for: ‘Fearful-place’ coding in the amygdala-hippocampal network
Source: eLife. 2021 Sep 17;10:e72040. doi: 10.7554/eLife.72040 (PMC8500711; doi:10.7554/eLife.72040)
Supplement: Supplementary file 1. — The percentage of the basal amygdala (BA) cell types. A total of 250 pyramidal cells were recorded. The bottom table shows the percentage of the cells showing the same, different, or no responses between the pre-robot and the robot sessions. Supplementary file 1B. Firing properties of dHPC place cells during the pre-robot, robot, and post-robot sessions. Kruskal–Wallis test (average firing rate, peak rate, spatial info, and running speed) and one-way ANOVA (field size); *p<0.05, **p<0.01, and ***p<0.001 compared to the nest cells; #p<0.05 compared to the proximal cells. Supplementary file 1C. Firing properties of place cells during the pre-stimulation, stimulation, and post-stimulation sessions. Data are from the rats with behavioral effects to the photostimulations. Kruskal–Wallis test; **p<0.01 and ***p<0.001 compared to the nest cells; #p<0.05, ##p<0.01, and ###p<0.001 compared to the proximal cells. Supplementary file 1D. Firing properties of place cells during the pre-stimulation, stimulation, and post-stimulation sessions. Data are from the rats without behavioral effects to the photostimulations. Kruskal–Wallis test; *p<0.05 and ***p<0.001 compared to the nest cells; #p<0.05 compared to the proximal cells. Supplementary file 1E. Descriptions and coordinates for each experiment. Supplementary file 1F. Summary of statistical tests performed. [file elife-72040-supp1.docx]

**Supplementary File 1A**

| **Cell type** | **Response type** | | **%** |
| --- | --- | --- | --- |
| **Robot cell** | | | |
|  | Excitation | | 45 (18) |
|  | Inhibition | | 2 (0.8) |
| **Pellet cell** | | | |
|  | Excitation | | 31 (12.4) |
|  | Inhibition | | 6 (2.4) |
| **Robot+Pellet** |  | | |
|  | Excitation + Excitation | | 17 (6.8) |
|  | Inhibition + Inhibition | | 2 (0.8) |
|  | Inhibition + Excitation | | 6 (2.4) |
| **Non-responsive** | | | |
|  |  | | 141 (56.4) |
| **Total** | | | 250 (100) |
|  |  |  |  |
| Same responses between the pre-robot and robot sessions | | | 7.6 |
| Different responses between the pre-robot and robot sessions | | | 36 |
| Non-responsive during the pre-robot and robot sessions | | | 56.4 |
| Total | | | 100 |

| **Properties** |  | **Pre-robot** | **Robot** | **Post-robot** |
| --- | --- | --- | --- | --- |
| Average firing rate (Hz) | Nest | 0.38 (0.17, 0.88) | 0.55 (0.30, 1.03) | 0.51 (0.28, 1.01) |
|  | Proximal | 0.46 (0.21, 0.70) | 0.51 (0.33, 1.14) | 0.39 (0.30, 1.14) |
|  | Distal | 0.39 (0.17, 0.67) | 0.52 (0.29, 0.89) | 0.57 (0.34, 0.94) |
| Field size (cm^2^) | Nest | 381.48 ± 14.32 | 468.59 ± 11.28 | 375.28 ± 13.28 |
|  | Proximal | 505.73 ± 39.60 * | 454.15 ± 30.45 | 417.15 ± 27.97 |
|  | Distal | 508.50 ± 23.36 *** | 537.86 ± 22.33 *** | 499.73 ± 21.98 *** |
| Peak rate (Hz) | Nest | 4.62 (2.35, 8.65) | 5.75 (2.99, 10.12) | 5.82 (2.60, 11.72) |
|  | Proximal | 5.43 (3.24, 13.25) | 5.09 (3.06, 10.68) | 5.89 (3.66, 12.45) |
|  | Distal | 5.31 (2.73, 10.07) | 5.27 (3.12, 11.21) | 7.19 (3.69, 12.74) |
| Spatial info (bits/s) | Nest | 10.18 (5.61, 21.63) | 7.67 (4.66, 11.72) | 10.32 (4.72, 20.53) |
|  | Proximal | 5.94 (3.58, 9.86) ** | 5.70 (2.91, 10.32) | 4.78 (2.75, 13.56) * |
|  | Distal | 3.91 (2.21, 7.75) *** | 3.84 (2.63, 7.41) *** | 4.07 (1.92, 7.90) *** |
| Running speed (cm/s) | Nest | 13.31 (12.72, 14.54) | 18.13 (16.61, 19.96) | 11.49 (10.30, 12.49) |
|  | Proximal | 13.66 (13.10, 14.40) | 16.52 (14.85, 18.13) ** | 11.26 (10.05, 12.27) |
|  | Distal | 14.28 (13.12, 15.02) * ^#^ | 18.42 (16.61, 19.48) ^#^ | 11.59 (10.78, 12.49) |

**Supplementary File 1B**

**Supplementary File 1C**

| **Properties** |  | **Pre-stimulation** | **Stimulation** | **Post-stimulation** |
| --- | --- | --- | --- | --- |
| Average firing rate (Hz) | Nest | 0.76 (0.39, 1.41) | 0.42 (0.23, 0.89) | 0.40 (0.13, 0.78) |
|  | Proximal | 0.59 (0.27, 1.36) | 0.43 (0.22, 0.72) | 0.26 (0.17, 0.94) |
|  | Distal | 0.58 (0.31, 1.01) | 0.52 (0.33, 1.13) | 0.34 (0.17, 0.78) |
| Field size (cm^2^) | Nest | 486 (378, 594) | 540 (423, 657) | 405 (288, 567) |
|  | Proximal | 450 (261, 567) | 477 (333, 567) | 396 (315, 522) |
|  | Distal | 567 (445.5, 688.5) ** ^#^ | 603 (477, 688.5) ^#^ | 540 (382.5, 679.5) *** ^#^ |
| Peak rate (Hz) | Nest | 6.03 (3.28, 11.37) | 4.94 (2.57, 9.56) | 4.39 (1.51, 7.31) |
|  | Proximal | 4.51 (1.71, 8.37) | 3.39 (2.19, 6.11) | 3.45 (1.53, 9.42) |
|  | Distal | 7.37 (3.55, 13.13) | 4.33 (2.54, 9.62) | 4.19 (2.03, 9.46) |
| Spatial info (bits/s) | Nest | 6.78 (4.00, 11.40) | 5.30 (3.45, 7.98) | 7.10 (4.25, 11.07) |
|  | Proximal | 7.12 (3.71, 14.74) | 5.71 (3.96, 10.22) | 6.87 (5.33, 12.99) |
|  | Distal | 2.62 (1.40, 5.16) *** ^###^ | 4.77 (3.38, 6.90) | 4.01 (2.18, 7.74) *** ^##^ |
| Running speed (cm/s) | Nest | 14.55 (12.66, 16.91) | 16.70 (12.83, 20.86) | 13.47 (11.04, 15.78) |
|  | Proximal | 15.86 (13.54, 17.95) | 17.06 (12.86, 22.53) | 13.47 (11.85, 15.78) |
|  | Distal | 15.41 (12.66, 16.26) | 17.06 (12.83, 21.82) | 13.47 (10.67, 16.61) |

**Supplementary File 1D**

| **Properties** |  | **Pre-stimulation** | **Stimulation** | **Post-stimulation** |
| --- | --- | --- | --- | --- |
| Average firing rate (Hz) | Nest | 0.55 (0.27, 0.97) | 0.62 (0.43, 1.55) | 0.70 (0.35, 1.17) |
|  | Proximal | 0.54 (0.26, 0.82) | 0.62 (0.50, 0.79) | 0.70 (0.52, 0.96) |
|  | Distal | 0.46 (0.34, 0.83) | 0.59 (0.30, 1.01) | 0.75 (0.35, 1.07) |
| Field size (cm^2^) | Nest | 342 (175, 504) | 400.5 (256.5, 571.5) | 423 (270, 603) |
|  | Proximal | 252 (189, 463) | 445.5 (272.25, 564.75) | 247.5 (229.5, 321.75) |
|  | Distal | 486 (400.5, 711) * | 594 (400.5, 675) | 558 (346.5, 720) ^#^ |
| Peak rate (Hz) | Nest | 4.03 (1.53, 6.41) | 7.26 (3.96, 9.50) | 7.40 (3.96, 9.50) |
|  | Proximal | 3.60 (2.59, 6.28) | 5.09 (2.41, 5.93) | 3.28 (2.28, 4.32) |
|  | Distal | 4.94 (3.47, 8.02) *** ^#^ | 5.06 (3.44, 9.00) | 4.95 (3.31, 11.42) ^#^ |
| Spatial info (bits/s) | Nest | 11.93 (5.64, 21.33) | 6.21 (3.34, 12.68) | 6.56 (3.37, 15.44) |
|  | Proximal | 17.39 (4.98, 23.18) | 12.02 (6.28, 19.04) | 23.96 (14.50, 35.41) |
|  | Distal | 2.83 (1.53, 5.96) | 3.19 (2.11, 6.37) | 3.84 (1.65, 8.15) |
| Running speed (cm/s) | Nest | 15.17 (14.02, 16.03) | 13.51 (13.14, 13.79) | 11.63 (11.40, 11.83) |
|  | Proximal | 15.72 (15.30, 15.80) | 13.79 (13.63, 13.96) | 11.83 (11.72, 12.22) |
|  | Distal | 14.61 (13.47, 15.72) | 13.73 (13.51, 13.79) | 11.83 (11.63, 12.73) |

**Supplementary File 1E**

| **Experiment descriptions** | **Figure No.** | **Dorsal hippocampus**  **(dHPC)** | | **Basal amygdala**  **(BA)** | |
| --- | --- | --- | --- | --- | --- |
|  |  | **Coordinates (from bregma)** | **Procedure** | **Coordinates**  **(from bregma)** | **Procedure** |
| Simultaneous recording from the dHPC and BA | Figure 1-2 | AP: -4.0 mm  ML: 2.0 mm  DV: -1.5 mm  (from dura) | Tetrodes implant | AP: -2.8 mm  ML: 5.0 mm  DV: -6.5 mm  (from dura) | Tetrodes implant |
| Optrode recording from the BA | Figure 3 *A-E* |  |  | AP: -2.8 mm,  ML: 5.0 mm (virus)  -5.2 mm (optrode)  DV: -8.8 mm (virus)  -5.5 mm (optrode from dura) | Virus injection, optrode implant |
| Optogenetic stimulation of the BA | Figure 3 *F-K* |  |  | AP: -2.8 mm  ML: ±5.0 mm  DV: -8.8 mm (virus)  8.4 mm (optic fiber) | Virus injection,  optic fiber implant |
| Optogenetic stimulation of the BA and place cell recording from the dHPC | Figure 4 | AP: -4.0 mm  ML: 2.0 mm  DV: -1.5 mm  (from dura) | Tetrodes implant | AP: -2.8 mm,  ML: 5.0 mm (unilateral)  ±5.0 mm (bilateral)  DV: -8.8 mm (virus)  8.4 mm (optic fiber) | Virus injection, optic fiber implant |

**Supplementary File 1F**

| **Figure** | | **Test** | **N** | **Statistics** | **P** | **Posttest** | **p (post)** |
| --- | --- | --- | --- | --- | --- | --- | --- |
| **Main figure statistics** | | | | | | | |
| 1 | B_left_ | One-way repeated-measures ANOVA | 32 recording days from 4 rats | *F_2, 62_* = 4089 | p<0.0001 | Bonferroni *post hoc* analysis | ****p<0.0001 |
|  | B_right_ | One-way repeated-measures ANOVA | 32 recording days from 4 rats | *F_2, 62_* = 27.21 | p<0.0001 | Bonferroni *post hoc* analysis | ****p<0.0001 |
| 2 | D_middle_ | Kruskal-Wallis test | Nest=213 cells  Proximal=25 cells  Distal=81 cells | *H* = 17.96 | p<0.0001 | Dunn’s multiple comparisons test | **p=0.0062  ***p=0.0003 |
|  | D_right_ | Kruskal-Wallis test | Nest=213 cells  Proximal=25 cells  Distal=81 cells | *H* = 23.54 | p<0.0001 | Dunn’s multiple comparisons test | **p=0.0047  ****p<0.0001 |
|  | G_nest+proximal_ | Two-tailed unpaired *t*-test | nonRobot cell-paired nest+proximal=29 cells  Robot cell paired nest+proximal=17 cells | *t*(44) = 0.2600 | p=0.7961 |  |  |
|  | G_distal_ | Two-tailed unpaired *t*-test | nonRobot cell-paired distal=15 cells  Robot cell paired distal=6 cells | *t*(19) = 2.797 | p=0.0119 |  |  |
|  | H_nest+proximal_ | Mann-Whitney test | nonRobot cell-paired nest+proximal=36 cells  Robot cell paired nest+proximal=17 cells | *U* = 624.5 | p=0.5939 |  |  |
|  | H_distal_ | Two-tailed unpaired *t*-test | nonRobot cell-paired distal=19 cells  Robot cell paired distal=15 cells | *t*(32) = 2.113 | p=0.0430 |  |  |
|  | I_first_ | Mann-Whitney test | Robot cell paired nest+proximal=19 cells  Robot cell paired distal=6 cells | *U* = 33.50 | p=0.1420 |  |  |
|  | I_second_ | Mann-Whitney test | nonRobot cell paired nest+proximal=30 cells  nonRobot cell paired distal=13 cells | *U* = 155 | p=0.2972 |  |  |
|  | I_third_ | Mann-Whitney test | Robot cell paired nest+proximal=23 cells  Robot cell paired distal=14 cells | *U* = 142.5 | p=0.5723 |  |  |
|  | I_fourth_ | Mann-Whitney test | Robot cell paired nest+proximal=51 cells  Robot cell paired distal=18 cells | *U* = 317 | p=0.2242 |  |  |
|  | J_left_ | Mann-Whitney test | Nest+proximal-paired Robot cells=13 cells  Distal-paired Robot cells=5 cells | *U* = 31.50 | p=0.9444 |  |  |
|  | J_right_ | Mann-Whitney test | Nest+proximal-paired Robot cells=17 cells  Distal-paired Robot cells=9 cells | *U* =68 | p=0.6723 |  |  |
| 3 | J_OFF_ | Two-tailed unpaired *t* test | EYFP=4  ChR2=5 | *t*(7) = 0.2845 | p=0.7843 |  |  |
|  | J_ON_75cm_ | Mann-Whitney test | EYFP=4  ChR2=5 | *U* =6 | p=0.0159 |  |  |
|  | J_ON_25cm_ | Mann-Whitney test | EYFP=4  ChR2=4 | *U* =0 | p=0.0286 |  |  |
|  | K | Two-way RM ANOVA | EYFP=4  ChR2=5 | Interaction effect  *F_72, 1908_* =  0.02540;  Trial effect *F_1.829, 12.80_* =  10.36;  Group effect *F_1,7_* = 0.025;  Subject effect *F_7,21_* = 5.497 | Interaction p=0.9944;  Trial p=0.0025;  Group p<8788;  Subject effect p=0.0011 |  |  |
| 4 | A | Friedman test | n=17 recording days | *χ²* = 32.72 | p<0.0001 | Dunn’s multiple comparisons test | Pre-stim vs. Stim: ****p<0.0001; Stim vs. Post-stim:  ****p<0.0001 |
|  | C_left_ | One-way ANOVA | Nest=107 cells  Proximal=21 cells  Distal=125 cells | *F_2, 250_* = 3.559 | p=0.031 | Bonferroni *post hoc* analysis | Nest vs. distal: *p=0.039 |
|  | C_right_ | Kruskal-Wallis | Nest=107 cells  Proximal=21 cells  Distal=125 cells | *H* = 8.019 | p=0.018 | Dunn’s multiple comparisons test | Nest vs. distal: *p=0.019 |
|  | E_nest_ | Friedman test | Nest=107 cells | *χ²* = 20.81 | p*<*0.001 | Dunn’s multiple comparisons test | Pre-stim vs. post-stim: ****p<0.0001;  Stim vs. post-stim: p=0.0028 |
|  | E_proximal_ | Friedman test | Proximal=21 cells | *χ²* = 1.300 | p=0.5220 |  |  |
|  | E_distal_ | Friedman test | Distal=125 cells | *χ²* = 10.70 | p=0.0048 | Dunn’s multiple comparisons test | Pre-stim vs. stim: **p=0.0063  Stim vs. post-stim: *p=0.0416 |
|  | F | Friedman test | n=6 recording days | *χ²* = 1.000 | p*>*0.999 |  |  |
|  | H_left_ | One-way ANOVA | Nest=30 cells  Proximal=8 cells  Distal=26 cells | *F_2, 68_* = 0.8399 | p=0.4362 |  |  |
|  | H_right_ | One-way ANOVA | Nest=30 cells  Proximal=8 cells  Distal=26 cells | *F_2, 68_* = 0.0512 | p=0.950 |  |  |
|  | J_nest_ | Friedman test | Nest=30 cells | *χ²* = 3.350 | p=0.1873 |  |  |
|  | J_proximal_ | Friedman test | Proximal=8 cells | *χ²* = 0.5806 | p=0.7642 |  |  |
|  | J_distal_ | Friedman test | Distal=26 cells | *χ²* = 1.806 | p=0.4054 |  |  |
| **Supplemental figure statistics** | | | | | | | |
| S1 | D_dHPC→BA_ | One-way repeated-measures ANOVA | N=40 | *F_2, 39_* = 28.86 | p<0.0001 | Bonferroni *post hoc* analysis | Pre-surge vs. post-surge: ****p<0.0001;  Pre-surge vs. pre-pellet:  **** p<0.0001 |
|  | D_BA→dHPC_ | One-way repeated-measures ANOVA | N=40 | *F_2, 39_* = 17.09 | p<0.0001 | Bonferroni *post hoc* analysis | Post-surge vs. pre-surge: ***p=0.0004;  Post-surge vs. post-pellet:  *** p=0.0001 |
|  | E_dHPC→BA_ | One-way repeated-measures ANOVA | N=59 | *F_2, 58_* = 37.08 | p<0.0001 | Bonferroni *post hoc* analysis | Pre-surge vs. post-surge: ****p<0.0001;  Pre-surge vs. pre-pellet:  **** p<0.0001 |
|  | E_BA→dHPC_ | One-way repeated-measures ANOVA | N=71 | *F_2, 70_* = 37.88 | p<0.0001 | Bonferroni *post hoc* analysis | Post-surge vs. pre-surge: ****p<0.0001;  Post-surge vs. post-pellet: ****p<0.0001 |
|  | F | Chi-squre | Pre-surge:  dHPC_leading_=40, BA_leading_=40;  Post-surge: dHPC_leading_=59,  BA_leading_=71 | *χ²* = 0.4234 | p=0.5153 |  |  |
| S2 | C | Two-way RM ANOVA | N=45 | Interaction effect:  *F_18, 1320_* = 13.99  Session effect:  *F_2, 1320_* =417.3  Time effect: *F_9, 1320_* = 21.94 | Interaction p*<*0.0001;  Time p*<*0.0001;  Group p*<*0.0001 | Bonferroni *post hoc* analysis | Pre-robot vs. robot: ****p<0.0001 for 0-6 s; ***p=0.0006 for 7 s; **p=0.0021 for 8 s;  Robot vs. post-robot:  ****p<0.0001 for 0-6 s; **p=0.0062 for 7 s; **p=0.0031 for 8 s; *p=0.0127  for 0 s |
| S3 | B_left_ | One-way ANOVA | Nest=213 cells  Proximal=25 cells  Distal=81 cells | *F_2, 316_* = 5.010 | p=0.007 | Bonferroni *post hoc* analysis | Nest vs. distal: *p=0.028;  Proximal vs. distal: *p=0.022 |
|  | B_right_ | One-way ANOVA | Nest=213 cells  Proximal=25 cells  Distal=81 cells | *F_2, 316_* = 1.749 | p=0.176 |  |  |
|  | C_left_ | One-way ANOVA | Nest=213 cells  Proximal=25 cells  Distal=81 cells | *F_2, 316_* = 3.269 | p=0.039 | Bonferroni *post hoc* analysis | Nest vs. proximal: *p=0.036 |
|  | C_right_ | One-way ANOVA | Nest=213 cells  Proximal=25 cells  Distal=81 cells | *F_2, 316_* = 7.383 | p=0.0007 | Bonferroni *post hoc* analysis | Nest vs. distal: **p=0.0011;  Proximal vs. distal: *p=0.0201 |
|  | D | One-way ANOVA | Nest=213 cells  Proximal=25 cells  Distal=81 cells | *F_2, 316_* = 3.714 | p=0.0254 | Bonferroni *post hoc* analysis | Nest vs. distal: *p=0.0103; |
|  | E | Two-way ANOVA | Nest=32 cells  Proximal=11 cells  Distal=16 cells |  | Interaction effect *F_4, 168_* = 0.7340;  Session effect *F_2, 168_* = 0.7782;  Group effect *F_2, 168_* = 12.05 | Interaction p*=*0.57;  Session p*=*0.4609;  Group p*<*0.0001 | 2. Nest vs. distal: ***p=0.0009;  3. Nest vs. distal: **p=0.0039 |
|  | H_left_ | One-way ANOVA | Nest=213 cells  Proximal=25 cells  Distal=81 cells | *F_2, 316_* = 7.477 | p=0.0007 | Bonferroni *post hoc* analysis | Nest vs. distal: **p=0.0017;  Proximal vs. distal: **p=0.0097 |
|  | H_middle_ | One-way ANOVA | Nest=213 cells  Proximal=25 cells  Distal=81 cells | *F_2, 316_* = 4.453 | p=0.0124 | Bonferroni *post hoc* analysis | Nest vs. distal: *p=0.0472;  Proximal vs. distal: *p=0.0304 |
|  | H_right_ | One-way ANOVA | Nest=213 cells  Proximal=25 cells  Distal=81 cells | *F_2, 316_* = 2.676 | p=0.070 |  |  |
|  | I | One-way repeated-measures ANOVA | N=32 | Treatment effect *F_3, 93_* = 5.660;  Individual effect *F_31, 93_* = 12.96 | Treatment effect p=0.0013; Individual effect p<0.0001 | Bonferroni *post hoc* analysis | 2 vs. 5: *p=0.0193;  2 vs. 10: **p=0.0012 |
| S4 | C_nest+proximal:pre-surge_ | Two-tailed unpaired *t*-test | nonRobot cell-paired nest+proximal=29 cells  Robot cell paired nest+proximal=17 cells | *t*(44) = 1.315 | p=0.1952 |  |  |
|  | C_distal:pre-surge_ | Two-tailed unpaired *t*-test | nonRobot cell-paired distal=15 cells  Robot cell paired distal=6 cells | *t*(19) = 0.9140 | p=0.3722 |  |  |
|  | C_nest+proximal:post-surge_ | Two-tailed unpaired *t*-test | nonRobot cell-paired nest+proximal=36 cells  Robot cell paired nest+proximal=17 cells | *t*(51) = 0.4426 | p=0.6599 |  |  |
|  | C_distal:post-surge_ | Two-tailed unpaired *t*-test | nonRobot cell-paired distal=19 cells  Robot cell paired distal=15 cells | *t*(32) = 2.825 | **p=0.0081 |  |  |
|  | D_left_ | Two-tailed unpaired t-test | Distal-nonRobot=16  Distal-Robot=12 | *t*(26) = 2.692 | p=0.0123 |  |  |
|  | D_right_ | Two-tailed unpaired t-test | nonRobot-distal=18  Robot-distal=9 | *t*(25) = 2.269 | p=0.0321 |  |  |
| S5 | C | Mann-Whitney test | Robot cells =25  nonRobot cells=44 | *U*=191.5 | p<0.0001 |  |  |
|  | D | Two-tailed unpaired t-test | Robot cell-paired nest+proximal cells =37  Robot cell-paired distal cells =17 | *t*(52) = 2.585 | p= 0.0126 |  |  |
|  | E_left_ | Paired t-test | N=8 | *t*(7) = 0.7607 | p= 0.4717 |  |  |
|  | E_right_ | Paired t-test | N=9 | *t*(8) = 0.6660 | p= 0.5241 |  |  |
|  | F | Paired t-test | N=9 | *t*(8) = 3.926 | p= 0.0044 |  |  |
|  | G_left_ | Paired t-test | N=7 | *t*(6) = 0.8892 | p= 0.4081 |  |  |
|  | G_right_ | Paired t-test | N=17 | *t*(16) = 1.356 | p= 0.1941 |  |  |
|  | H | Paired t-test | N=13 | *t*(12) = 4.593 | p= 0.0006 |  |  |
| S7 | D_left_ | One-way ANOVA | Nest=107 cells  Proximal=21 cells  Distal=125 cells | *F_2, 250_* = 3.517 | p=0.0312 | Bonferroni *post hoc* analysis | Nest vs. distal: **p=0.0350 |
|  | D_right_ | Kruskal-Wallis | Nest=107 cells  Proximal=21 cells  Distal=125 cells | *H* = 4.832 | p=0.089 |  |  |
|  | E_left_ | Kruskal-Wallis | Nest=107 cells  Proximal=21 cells  Distal=125 cells | *H* = 7.646 | p=0.022 | Dunn’s multiple comparisons test | Nest vs. proximal: *p=0.021  Proximal vs. distal: *p=0.026 |
|  | E_right_ | Kruskal-Wallis | Nest=107 cells  Proximal=21 cells  Distal=125 cells | *H* = 10.64 | p=0.0049 | Dunn’s multiple comparisons test | Proximal vs. distal: *p=0.0110 |
|  | G | Kruskal-Wallis | Nest=107 cells  Proximal=21 cells  Distal=125 cells | *H* = 1.829 | p=0.4007 |  |  |
|  | H | One-way ANOVA | Nest_neutral_=114  Nest_excited_=7  Nest_inhibited_=4 | *F_2, 122_* = 0.398 | p=0.6725 |  |  |
|  |  | One-way ANOVA | Distal_neutral_=93  Distal_excited_=10  Distal_inhibited_=4 | *F_2, 104_* = 0.712 | p=0.4930 |  |  |
|  | K_left_ | One-way ANOVA | Nest=30 cells  Proximal=8 cells  Distal=26 cells | *F_2, 61_* = 0.5627 | p=0.5726 |  |  |
|  | K_right_ | Kruskal-Wallis | Nest=30 cells  Proximal=8 cells  Distal=26 cells | *H* = 0.9559 | p=0.620 |  |  |
|  | L_left_ | One-way ANOVA | Nest=30 cells  Proximal=8 cells  Distal=26 cells | *F_2, 61_* = 1.752 | p=0.182 |  |  |
|  | L_right_ | Kruskal-Wallis | Nest=30 cells  Proximal=8 cells  Distal=26 cells | *H* = 6.288 | p=0.0431 | Dunn’s multiple comparisons test | Proximal vs. distal: *p=0.0469 |
